# Carbetocin in comparison with oxytocin in several dosing regimens for the prevention of uterine atony after elective caesarean section in the Netherlands

**DOI:** 10.1007/s00404-012-2693-8

**Published:** 2013-01-18

**Authors:** C. A. G. Holleboom, J. van Eyck, S. V. Koenen, I. A. M. Kreuwel, F. Bergwerff, E. C. Creutzberg, H. W. Bruinse

**Affiliations:** 1 Departments of Gynaecology, Bronovo Hospital, The Hague, The Netherlands; 2 Departments of Gynaecology, Isala Clinics, Zwolle, The Netherlands; 3 Departments of Gynaecology, University Medical Centre, Utrecht, The Netherlands; 4 Departments of Gynaecology, Medical Spectrum Twente, Enschede, The Netherlands; 5 Departments of Gynaecology, St. Antonius Hospital, Nieuwegein, The Netherlands; 6 Departments of Gynaecology, Ferring B.V. Hoofddorp, P.O. Box 184, 2130 AD Hoofddorp, The Netherlands

**Keywords:** Carbetocin, Oxytocin, Caesarean section, Uterine atony, Post-partum haemorrhage, Blood transfusion

## Abstract

**Purpose:**

The aim of the study was to compare the prophylactic effects of carbetocin with those of oxytocin for the prevention of uterine atony in patients undergoing elective caesarean section (CS) in the Netherlands. The primary endpoint was the need for additional uterotonic medication.

**Methods:**

Each of the five participating Dutch hospitals treated 50–100 term patients with 100 μg of intravenous carbetocin on prescription. Each centre retrieved charts of 250 patients treated with oxytocin according to the hospital’s policy for the prevention of uterine atony (oxytocin bolus 5 IU, bolus 10 IU or bolus 5 IU followed by 10 IU in 2 h).

**Results:**

In the carbetocin group 462 subjects were included and in the oxytocin group 1,122. The proportion of subjects needing additional uterotonic treatment was 3.1 % (95 % CI 1.7–5.1 %) after carbetocin and 7.2 % (5.8–8.9 %) after oxytocin; relative risk 0.41 (0.19–0.85); *p* = 0.0110. Carbetocin was most effective compared with the oxytocin 5 IU bolus subgroup with less need for additional uterotonic medication (3.1 vs. 9.3 %, *p* = 0.0067) and blood transfusions (2.2 vs. 3.6 %, *p* = 0.0357).

**Conclusions:**

Compared with oxytocin, prophylaxis of uterine atony with carbetocin after an elective CS diminished the need for additional uterotonics by more than 50 %.

## Introduction

Postpartum haemorrhage (PPH) is defined as a blood loss >500 ml and serious PPH as a blood loss >1,000 ml. PPH is a serious condition remaining the single main cause of maternal morbidity and mortality [1]. In the Netherlands, 1 out of 14 labouring women experiences PPH [2]. The most frequent cause of PPH is uterine atony, contributing up to 80 % of the PPH cases [3]. Although two-thirds of the PPH cases occur in women without predisposing factors, there are several risk factors for PPH such as previous PPH, pre-eclampsia, coagulopathy, multiple gestation and antepartum haemorrhage. Also caesarean section (CS) is a recognised risk factor for PPH and its prevalence is increasing [4, 5]. In the Netherlands the number of births is around 180,000 per year, of which 15 % are CS.

The impact of PPH on maternal morbidity and mortality makes active management of the third stage of labour to a critical key [5, 6]. To this end, uterotonic agents are administrated immediately after delivery of the baby [3].

Oxytocin (Syntocinon^®^) is currently the uterotonic of first choice. It has proven to decrease the incidence of PPH by 40 % and has a rapid onset of action and a good safety profile [3, 7–9]. A disadvantage of oxytocin is its short half-life of 4–10 min, regularly requiring a continuous intravenous infusion or repeated intramuscular injections [10].

Carbetocin (Pabal^®^) is a long-acting oxytocin analogue indicated for the prevention of uterine atony after child birth by CS under epidural or spinal anaesthesia. Carbetocin has a rapid onset of action (within 1–2 min) and a prolonged duration of action (approximately 1 h) because of sustained uterine response with contractions of higher amplitude and frequency. Its safety profile is comparable to that of oxytocin [11].

The current pharmacological policy for the prevention of PPH in the Netherlands is oxytocin [3]. Most hospitals use a bolus of oxytocin 5 or 10 IU; some add an infusion of oxytocin for a couple of hours. The aim of the study was to compare the prophylactic effects of carbetocin with those of oxytocin in several dosing regimens for the prevention of uterine atony in patients undergoing elective CS under regional anaesthesia in the Netherlands.

## Methods

### Design

The study had an open-label, multi-centre, prospective combined with retrospective, observational design. From January 2009 until October 2010 each of the five centres treated 50–100 term patients with carbetocin. In the same time period, each centre retrieved charts of 250 patients treated with oxytocin according to the hospital’s policy for the prevention of uterine atony. In three hospitals an oxytocin bolus 5 IU was routinely administered, in one hospital an oxytocin bolus 10 IU and in one hospital an oxytocin bolus 5 IU followed by an infusion with 10 IU in 2 h.

### Patients

Term patients (gestational age ≥37 weeks) undergoing an elective CS under epidural and/or spinal anaesthesia were treated on physician’s prescription with a single injection of 100 μg of intravenous carbetocin in accordance with the summary of product characteristics (SmPC). Data of patients treated with carbetocin after elective CS under regional anaesthesia in a previous observational study were merged (140 observations) [12]. An elective CS was defined as a CS where there was no intention for vaginal delivery (f.i. breech presentation, CS in medical history or uterine scars).

No extra measurements were performed, other than the procedures routinely performed during CS. No distinction towards risk factors was made. If needed at the discretion of the obstetrician, patients were treated subsequently according to the hospital’s guidelines with additional uterotonics, blood transfusions or (surgical) interventions.

### Data collection

Patient demographics, medical history and information on the current pregnancy, co-morbidities and co-medication were collected by means of web-based data entry application (WBDEA). The study focuses on early or primary PPH, i.e. blood loss during the first 24 h after delivery [13]. During this time span, information on additional uterotonic medication, blood transfusion, operative interventions related to PPH, haemoglobin and haematocrit (only if routinely assessed) and estimated intra-operative blood loss was collected. Also unsolicited adverse events (AEs) were noted.

The following parameters were only documented in the prospective group: position of fundus after wound closure, uterine tone, need for uterine massage and the subjective experience of the doctor with carbetocin.

### Endpoints

Primary endpoint was the need for additional uterotonic medication after carbetocin or oxytocin administration. Secondary endpoints included the need for blood transfusion or operative interventions related to PPH, the change in haemoglobin and haematocrit post versus pre CS, the estimated amount of intra-operative blood loss and the incidence of (serious) PPH (blood loss >500 and >1,000 ml).

### Statistics

The aimed number of patients treated with carbetocin was 500. This group was compared with an aimed 1,250 patients treated with oxytocin. On the base of the study of Dansereau et al. [14], the power of this comparison is 98 %. The different oxytocin regimens allowed us to make substratifications in the oxytocin arm.

Total group results were presented as mean (standard deviation; SD), median (range) or, for the dichotomous variables, as relative proportions. Differences between the groups were analysed by the Mantel–Haenszel test with stratification for centre. To investigate the contributing factors to the need for additional uterotonic medication, logistic regression with the following factors was performed: centre, gestational age, age, BMI (body mass index), ethnicity, smoking/alcohol use, parity, PPH in medical history, antepartum blood loss and multiple pregnancy. Significance was determined at the level of 5 %.

## Results

### Disposition of subjects

In the carbetocin group 462 subjects were included and in the oxytocin group 1,122 (oxytocin bolus 5 IU *n* = 690, oxytocin bolus 10 IU *n* = 248; oxytocin bolus 5 IU followed by 10 IU in 2 h *n* = 172). For 12 subjects, oxytocin dose was missing; these cases were only taken into account in the total oxytocin group.

### Patient characteristics

In Table 1, patient’s demographics, medical history and information on current pregnancy are shown for the carbetocin and oxytocin treated patients. There were no statistically significant differences between the groups.

**Table 1:** Patient’s demographics, medical history and information on current pregnancy

	Carbetocin	Oxytocin	*p* value
*n*	462	1,122	
Age (years); mean (SD)	33.0 (4.6)	33.3 (4.6)	NS
Usual BMI (body mass index) before pregnancy (kg/m^2^); mean (SD)	26.0 (5.4)	25.5 (5.0)	NS
% Caucasian women	81.2	78.0	NS
% women with 0, 1 or 2 + living births	27.3, 44.4, 28.3	31.8, 45.1, 23.1	NS
% patients with repeated C. section	59.0	48.6	NS
% patients with known fluxus postpartum in medical history	7.9	5.2	NS
Gestational age (weeks)	38.9 (1.0)	38.8 (1.0)	NS
% patients with myoma	3.8	2.6	NS
% patients with gestational hypertension	4.8	6.3	NS
% patients with gestational diabetes	4.6	6.0	NS
% patients with (pre-) eclampsia or HELLP syndrome	2.2	3.7	NS
% patients with abnormal placental position	3.9	5.1	NS
% patients with antepartum blood loss	3.9	3.9	NS
% patients with multiple pregnancy	3.5	2.1	NS
Birth weight of child (g); mean (SD)	3,514 (498)	3,420 (545)	NS
% patients with manual removal of placenta (vs umbilical cord traction)	66.3	64.6	NS
% patients with co-morbidity	16.0	21.5	NS
% patients with co-medication	20.6	14.6	NS

### Primary endpoint: need for additional uterotonic medication

The proportion of subjects needing additional uterotonic treatment was 3.1 % (95 % confidence interval (CI) 1.7–5.1 %) in the carbetocin group and 7.2 % (95 % CI 5.8–8.9 %) in all oxytocin groups combined, *p* = 0.0110 (Table 2). The relative risk for additional uterotonic treatment after carbetocin versus oxytocin was 0.41 (95 % CI 0.19–0.85).

**Table 2:** Percentage of subjects needing additional uterotonic treatment per centre

	*n*	% (95% CI)	Centre (%)					
			1	2	3	4	5	6
Carbetocin	462	3.1 (1.7–5.1)	1.8	3.9	2.8	2.2	2.4	4.5
Oxytocin total	1,122	7.2 (5.8–8.9)	0.0	16.2	9.5	6.0	1.8	
Oxytocin 5 IU	690	9.3 (7.2–11.7)		16.1	9.2		1.8	
Oxytocin 5 IU + infusion	172	0.0	0.0					
Oxytocin 10 IU	248	6.5 (3.7–10.3)		20		6.2		
Oxytocin dose missing	12							

Centres:* 1* Isala Clinics, *2* UMC Utrecht, *3* Bronovo Hospital, *4* St. Antonius Hospital, *5* Medical Spectrum Twente, *6* Centres from previous observational carbetocin study [12]

The need for additional uterotonics was significantly lower after carbetocin versus the subgroup oxytocin 5 IU (3.1 vs. 9.3 %, *p* = 0.0067), but not different from the subgroups oxytocin 10 IU (6.5 %) or oxytocin 5 IU + infusion (0.0 %) (Table 3). A significant difference between the centres in observed effects was seen (*p* = 0.0368) (Table 3).

**Table 3:** Need for additional uterotonic treatment (stratified Mantel–Haenszel test)

Comparison	*p* value	Relative risk (CI)	Breslow day test (*p* value)
Carbetocin versus oxytocin total	0.0110	0.41 (0.19–0.85)	0.0368
Carbetocin versus oxytocin 5 IU	0.0067	0.32 (0.13–0.79)	0.3223
Carbetocin versus oxytocin 5 IU + infusion	0.0791	Indefinable	Indefinable
Carbetocin versus oxytocin 10 IU	0.1300	0.31 (0.06–1.56)	0.6453

Additional uterotonic treatment was most often oxytocin infusion, occasionally prostaglandins and only once methergin.

On logistic regression, the following factors contributed to the need for additional uterotonic medication: treatment with oxytocin versus carbetocin (*p* = 0.0049), antepartum blood loss (*p* = 0.0057) and multiple pregnancy (*p* = 0.0111).

### Secondary endpoints

Blood transfusions were administrated in 2.2 % of the cases in the carbetocin group and 2.7 % in the oxytocin group (NS). The subgroup oxytocin 5 IU had a significant higher incidence of blood transfusions (3.6 %) compared with the carbetocin group (*p* = 0.0357), while the 5 IU oxytocin + infusion group (0 %) and the oxytocin 10 IU group (1.6 %) had significant lower incidences of blood transfusions than the carbetocin group (*p* = 0.0312 and *p* = 0.0451, respectively).

Mean number of packed cells per patient was 0.04 versus 0.09 in the carbetocin and oxytocin groups, respectively (*p* = 0.129). Looking at subgroups, the mean number of packed cells per patient was lower in the carbetocin group than in the oxytocin 5 IU group (0.04 vs. 0.12, *p* = 0.024), but not different from the 5 IU oxytocin + infusion group (0.0, *p* = 0.167) and the oxytocin 10 IU group (0.04, *p* = 1). However, for three patients in the carbetocin group, the number of packed cells was unknown. For those cases, the statistician filled in the most frequent value (2 packed cells). This resulted in the following figures: carbetocin versus oxytocin: 0.05 versus 0.09, *p* = 0.277 and carbetocin versus oxytocin 5 IU: 0.05 versus 0.12, *p* = 0.063.

The need for additional operative interventions related to PPH (i.e. uterine tamponade, ligation of vessels or relaparotomy) tended to be lower in the carbetocin group than in the oxytocin group (0.4 % vs. 1.2 %, *p* = 0.0737). No significant differences were seen between carbetocin and the subgroups oxytocin 5 IU, oxytocin 5 IU + infusion and oxytocin 10 IU (1.9, 0.0 and 0.4 %, respectively).

The changes in haemoglobin, haematocrit and estimated peri-operative blood loss were similar between the groups (Table 4). The proportion of subjects with blood loss >500 ml (carbetocin 28.8 %, oxytocin 26.9 %) and >1,000 ml (carbetocin 7.8 %, oxytocin 8.4 %) was also comparable for both groups.

**Table 4:** Changes in haemoglobin and haematocrit and estimated peri-operative blood loss

	Change in Hb^a^	Change in Hb^a^	Change in Ht^b^	Change in Ht^b^	Blood loss	Blood loss
	Mean (SD)	Median	Mean (SD)	Median	Mean (SD)	Median (range)
Carbetocin	−0.87 (0.57)	−0.80	−0.035 (0.046)	−0.030	539 (424)	400 (0–5,000)
Oxytocin total	−0.94 (0.62)	−0.90	−0.046 (0.041)	−0.040	536 (496)	400 (0–6,000)
Oxytocin 5 IU	−1.01 (0.62)	−1.00	−0.050 (0.046)	−0.050	588 (570)	400 (0–6,000)
Oxytocin 5 IU + infusion	−0.93 (0.58)	−0.90	−0.042 (0.029)	−0.040	468 (227)	400 (200–2,000)
Oxytocin 10 IU	−0.86 (0.62)	−0.80	−0.042 (0.038)	−0.040	434 (374)	350 (0–3,284)

*Hb* haemoglobin (mmol/l), *Ht* haematocrit (l/l), *blood loss* estimated peri-operative blood loss (ml)

^a^Carbetocin: *n* = 279; oxytocin: *n* = 652

^b^Carbetocin: *n* = 230; oxytocin: *n* = 538

After carbetocin, the need for uterine massage was 3.4 %. The fundus uteri was at or below the umbilicus in 92.9 % of the patients and uterine tone was firm in 97.1 %. Physician’s subjective experience with carbetocin was rated as good in 92 % of the cases.

### Adverse events

In the carbetocin group a total of 11 AEs were experienced by nine subjects. Three subjects experienced a serious AE (SAE). In two cases, the SAE was life-threatening (“retroperitoneal haemorrhage’’ and “haemorrhage’’) but the relationship with carbetocin was rated as unlikely. The third case, “decrease in blood pressure’’, was considered as an important medical event and rated as probably related to carbetocin.

Two subjects experienced at least one AE with a moderate degree of severity and four subjects experienced a mild AE after carbetocin (a.o. nausea, headache, abdominal pain). In those six cases, the relationship with carbetocin was rated as unlikely or none.

In the oxytocin group a total of six AEs were experienced by six subjects. Two subjects experienced moderate AEs (fluxus, atony) unrelated to oxytocin. Four subjects experienced mild AEs like hypotension or fluxus, rated as having no, unlike or possible relationship with oxytocin.

## Discussion

The present study showed that the need for additional uterotonics after an elective CS under regional anaesthesia was more than halved after prevention of uterine atony with 100 μg intravenous carbetocin compared with intravenous oxytocin in several dosing regimens (3.1 % vs. 7.2 %). Estimated peri-operative blood loss, changes in haemoglobin and haematocrit as well as the need for blood transfusions or additional operative interventions were not different between carbetocin and oxytocin.

Carbetocin seemed to be most beneficial compared with the subgroup oxytocin 5 IU bolus with thrice less need for additional uterotonic medication (3.1 vs. 9.3 %) and significantly less need for blood transfusions (2.2 vs. 3.6 %).

Carbetocin led to a prompt and sustained uterine involution with firm uterine tone. The majority of the doctors was satisfied with the use of carbetocin.

To our knowledge, this is the first study that compared carbetocin with oxytocin in several dosing regimens used in the Netherlands for the prevention of uterine atony. The results are in line with those of the four existing clinical studies comparing carbetocin with oxytocin in CS. Compared with intravenous oxytocin administration for several hours, carbetocin resulted in a more rapid and sustained uterine involution [15, 16], less need for additional uterotonic medication [14, 15], longer median time to intervention [14], less need for uterine massage [15] and more often mild blood loss (<200/<500 ml) [15, 16]. Compared with a single bolus of oxytocin 5 IU, PPH prevention with carbetocin resulted in less need for additional uterotonic medication [17].

Since all previous studies as well as the present study demonstrated a lower rate of additional oxytocic usage after carbetocin compared with oxytocin, carbetocin maybe more effective in preventing uterus atony and thereby PPH. On base of the recently updated Cochrane review the difference in risk for PPH after CS (defined as blood loss >500 ml or as defined by trialist) between carbetocin and oxytocin did not reach statistical significance (risk ratio (RR) 0.66; 95 % confidence interval (CI) 0.42–1.06, *p* = 0.086). Use of carbetocin did result in a reduction in the need for therapeutic uterotonics (RR 0.62; 95 % CI 0.44–0.88) as well as the need for uterine massage (RR 0.54; 95 % CI 0.37–0.79) after CS [18].

The lesser need for additional uterotonics after carbetocin versus oxytocin was not reflected in a lower amount of peri-operative blood loss in the carbetocin group. However, this parameter was estimated by the surgeon and not calculated by counting used swabs or sensitive colorimetry [15] and may have been imprecise. In the study of Borruto, carbetocin versus oxytocin infusion (10 IU in 2 h) resulted in a lower proportion of patients with blood loss >500 ml (19 vs. 45 %, *p* = 0.05) after CS in patients with at least one risk factor for PPH [15].

There was no difference in the postoperative drop in haemoglobin and haematocrit, but these values were only recorded if assessed during routine care. Therefore, results may be biased due to measurements in selected patients.

In addition to treatment with oxytocin or carbetocin, the factors ‘antepartum haemorrhage’ and ‘multiple pregnancy’ had an independent and significant impact on the need for additional uterotonics. These are indeed known risk factors for PPH [4, 5].

The need for additional uterotonics indicates a diagnosis of uterine atony which implies intensified monitoring and possibly prolonged observation time in the postoperative recovery area or labour ward with an increased use of medical staff time. The lesser use of additional oxytocics after carbetocin found in this study is therefore an important surrogate outcome measure with possible financial savings. To date, two studies have compared the cost-effectiveness of prophylactic carbetocin and oxytocin following CS. In low-risk women undergoing elective CS, carbetocin was not cost saving [19]. In contrast, in patients with risk factors for PPH, the mean cost per woman was significantly lower following carbetocin treatment compared with oxytocin treatment [20].

In addition to effectivity, the administration of a single injection of carbetocin is more convenient than an oxytocin bolus injection that needs to be followed by several hours of oxytocin infusion. The latter requires preparation of a dosing pump and is therefore more prone to (dosing) errors [21].

The present study did not demonstrate differences in the need for additional uterotonics after carbetocin versus oxytocin 5 IU bolus followed by an oxytocin infusion or oxytocin bolus 10 IU. It must be taken into account that analysis results specific for these two subgroups could not be distinguished from centre effects and the effects found should be regarded in that perspective.

Considering oxytocin bolus 5 IU + infusion, a recent study demonstrated that the need for an additional uterotonic agent after oxytocin bolus 5 IU followed by 40 IU oxytocin infusion over 4 h was lower than that in the oxytocin bolus 5 IU only group [22]. A bolus of oxytocin 10 IU could theoretically have more cardiovascular side effects compared with a bolus of oxytocin 5 IU [23] or carbetocin [17].

Data on carbetocin were prospectively collected while data on oxytocin were retrospectively collected; therefore, a possible underreporting in the retrospective group cannot be excluded. Furthermore, some retrospective data could possibly be better traced than other (for instance, need for additional treatments versus uterine massage or adverse events).

Nevertheless, we chose the design of the study to be observational because of the following reasons: it allows collecting ‘real life’ data obtained in an unselected patient population according local treatment routines and protocols. As a result an observational design reflects more closely the real clinical situation rather than a randomised clinical trial design would have done [24]. Only cases with elective CS were included in the study, to minimise variation in the outcome measures due to other factors. The observational design allowed us to recruit a rather large number of patients in a limited time frame. The cohort was large enough to make in addition comparisons between carbetocin and oxytocin subgroups. Finally, we considered that it was not necessary to add another RCT to the properly performed RCTs already published on carbetocin versus oxytocin.

The incidence and nature of AEs was in line with the SmPCs of carbetocin and oxytocin and the adverse effect profiles appeared reassuringly similar between the two medications. Recent studies show that carbetocin and oxytocin have comparable (transient) haemodynamic effects and both drugs have the same acceptable safety profile [11, 17].

In conclusion, compared with oxytocin in several dosing regimens, this study demonstrates that prophylaxis of uterine atony with carbetocin after an elective CS reduced the need for additional uterotonics by more than 50 %.
